# PD110 Ward Round Woes: Accuracy Of Ward Round Documentation

**DOI:** 10.1017/S0266462324003520

**Published:** 2025-01-07

**Authors:** Ellie Treloar, Ying Y. Ting, Martin Bruening, Jessica Reid, Suzanne Edwards, Emma L. Bradshaw, Jesse D. Ey, Matthias Wichmann, Matheesha Herath, Guy J. Maddern

## Abstract

**Introduction:**

Ward round quality is a pivotal component of surgical care and is intimately associated with patient outcomes. Despite this, ward rounds remain largely understudied and underrepresented in medical literature. Accurate and thorough ward round documentation is known to improve communication and patient outcomes and to reduce hospital expenditure. This study aimed to determine the accuracy of ward round documentation.

**Methods:**

A prospective observational cohort study was performed as a sub-analysis of a larger study by reviewing 135 audiovisual recordings of surgical ward rounds over two years at two hospitals. The recordings were transcribed verbatim, and content was designated a level of importance by an external reviewer. This was then compared to the written case notes to determine the accuracy and importance of omitted documentation. Patient age, sex, and length of stay, as well as the senior doctor leading and the intern documenting the ward round, were assessed using multivariable linear mixed-effect models to determine their impact on documentation accuracy.

**Results:**

Nearly one-third (32.4%) of spoken information on the surgical ward round that was deemed “important”, including discharge plans and bookings for surgery, was absent from the patients’ electronic medical records. Additionally, in 11 percent of case notes there was a major conflict between the ward round discussion and what was documented. Younger patients (p=0.04) and patients who had been on the ward longer (p=0.005) were less likely to have accurate documentation. Some interns were significantly worse at documenting discussions than were others (p<0.0001). Day of the week, location, and the senior doctor present did not affect documentation accuracy.

**Conclusions:**

This study demonstrates that a significant amount of important discussion during surgical ward rounds regarding patient care is not recorded accurately, or at all, in the patient medical record. This can lead to preventable patient complications and longer hospital stays, resulting in increased strain on hospital resources. This study emphasizes the need for further research to address this problem.

